# Obesity-related known and candidate SNP markers can significantly change affinity of TATA-binding protein for human gene promoters

**DOI:** 10.1186/1471-2164-16-S13-S5

**Published:** 2015-12-16

**Authors:** Olga V Arkova, Mikhail P Ponomarenko, Dmitry A Rasskazov, Irina A Drachkova, Tatjana V Arshinova, Petr M Ponomarenko, Ludmila K Savinkova, Nikolay A Kolchanov

**Affiliations:** 1 Institute of Cytology and Genetics, Siberian Branch of Russian Academy of Sciences, 10 Lavrentyeva Avenue, Novosibirsk 630090, Russia; 2 Novosibirsk State University, 2 Pirogova Street, Novosibirsk 630090, Russia; 3 Children's Hospital Los Angeles, 4640 Hollywood Boulevard, University of Southern California, Los Angeles, CA 90027, USA; 4 Laboratory of Evolutionary Bioinformatics and Theoretical Genetics, Institute of Cytology and Genetics, Siberian Branch of Russian Academy of Sciences, 10 Lavrentyev Avenue, Novosibirsk 630090, Russia

**Keywords:** Obesity, gene, promoter, TATA-binding protein, TBP, single nucleotide polymorphism, SNP, TBP/promoter affinity change, statistical significance, candidate SNP marker, prediction *in silico*, verification *in vitro*

## Abstract

**Background:**

Obesity affects quality of life and life expectancy and is associated with cardiovascular disorders, cancer, diabetes, reproductive disorders in women, prostate diseases in men, and congenital anomalies in children. The use of single nucleotide polymorphism (SNP) markers of diseases and drug responses (i.e., significant differences of personal genomes of patients from the reference human genome) can help physicians to improve treatment. Clinical research can validate SNP markers via genotyping of patients and demonstration that SNP alleles are significantly more frequent in patients than in healthy people. The search for biomedical SNP markers of interest can be accelerated by computer-based analysis of hundreds of millions of SNPs in the 1000 Genomes project because of selection of the most meaningful candidate SNP markers and elimination of neutral SNPs.

**Results:**

We cross-validated the output of two computer-based methods: DNA sequence analysis using Web service SNP_TATA_Comparator and keyword search for articles on comorbidities of obesity. Near the sites binding to TATA-binding protein (TBP) in human gene promoters, we found 22 obesity-related candidate SNP markers, including rs10895068 (male breast cancer in obesity); rs35036378 (reduced risk of obesity after ovariectomy); rs201739205 (reduced risk of obesity-related cancers due to weight loss by diet/exercise in obese postmenopausal women); rs183433761 (obesity resistance during a high-fat diet); rs367732974 and rs549591993 (both: cardiovascular complications in obese patients with type 2 diabetes mellitus); rs200487063 and rs34104384 (both: obesity-caused hypertension); rs35518301, rs72661131, and rs562962093 (all: obesity); and rs397509430, rs33980857, rs34598529, rs33931746, rs33981098, rs34500389, rs63750953, rs281864525, rs35518301, and rs34166473 (all: chronic inflammation in comorbidities of obesity). Using an electrophoretic mobility shift assay under nonequilibrium conditions, we empirically validated the statistical significance (α < 0.00025) of the differences in TBP affinity values between the minor and ancestral alleles of 4 out of the 22 SNPs: rs200487063, rs201381696, rs34104384, and rs183433761. We also measured half-life (t_1/2_), Gibbs free energy change (ΔG), and the association and dissociation rate constants, k_a _and k_d_, of the TBP-DNA complex for these SNPs.

**Conclusions:**

Validation of the 22 candidate SNP markers by proper clinical protocols appears to have a strong rationale and may advance postgenomic predictive preventive personalized medicine.

## Background

Metabolic syndrome, one of the main global challenges for modern health care [1], involves elevated risk of several interrelated disorders: obesity, ischemic heart disease, hypertension, insulin resistance, type II diabetes mellitus, and dyslipidemia. It is commonly believed that obesity is among the key risk factors of metabolic syndrome [2]. Obesity affects quality of life and life expectancy and is associated with cardiovascular disorders [3] (such as hypertension [6,7]), cancer [4,5], diabetes [8,9], and damage to kidneys and liver [10]. It has also been reported that obesity correlates with reproductive disorders in women [11-13], prostate diseases in men [14], and elevated risk of congenital anomalies in children [15].

The discovery [16] that the leptin gene *LEP *is "the obesity gene" (*OB; *i.e., *LEP ≡ OB) *shed light on the regulation of energy metabolism [17]. Leptin is a peptide hormone with molecular mass ~16 kD [18]. Its secretion by adipocytes is directly proportional to adipose-tissue weight. Recessive mutations in the leptin gene are strongly associated with obesity in mice and humans [19]. Leptin circulates with blood and regulates food consumption and energy demand of the brain. With a decrease in fat weight, the plasma leptin level decreases to increase appetite and suppress energy consumption until the recovery of fat weight. When fat weight increases, the level of circulating leptin increases and suppresses appetite until a fat weight reduction [19]. Initially, it was believed that leptin is produced mainly by adipocytes [20], but later it was found that leptin is also produced in such organs as the stomach [21], heart [22], and placenta [23]. In addition to body weight control, leptin plays other physiological roles. It modulates vascular tone and blood pressure and enhances angiogenesis and calcification of vascular cells [22-24]. Numerous studies showed a direct correlation between blood leptin concentrations and blood pressure [25]. Elevated blood pressure contributes to atherosclerosis [26]. Recent studies on the development of obesity in male Wistar rats uncovered an association of diet-induced fatty liver (steatosis) with insulin/leptin resistance [27].

Recent years have witnessed an increasing number of women with metabolic imbalance during pregnancy, with a variety of consequences; the mechanism of this metabolic imbalance is still poorly understood [28]. The molecular pathways linking obesity to the above disorders are unclear [29] mostly because of the large number of genes related to the nervous system, endocrine system, and metabolic system that regulate energy metabolism. The contribution of a single gene is estimated to be 1-6% [14], which is close to the statistical significance threshold with the current accuracy of these estimates. Postgenomic predictive preventive medicine [30] offers a way around this obstacle (poor understanding of the pathogenesis of obesity as a whole) by taking into account numerous SNP markers of various partially obesity-related complications in the above-mentioned or other disorders [31,32].

Analysis of SNP markers, which show differences between an individual human genome and the reference human genome (hg19), as part of postgenomic preventive personalized medicine allows for effective treatment [33], improvement of treatment [34], and prevention of complications of treatment [35]. Genome-wide SNP identification is the goal of the project 1000 Genomes [36]. The dbSNP database [37] documents current results of this project [36]; thus, the reference human genome, hg19, which is thought to contain the ancestral versions of all SNPs, is constantly refined. It is available in the Ensembl database [38] via the Web service UCSC Genome Browser [39]. Ensembl contains data on gene knockouts in animals, and this information is helpful, for example, for reconstruction of perturbation networks of various disorders and for development of therapeutic strategies [40].

Computer-based analysis of hundreds of millions of unannotated SNPs in 1000 Genomes [36] that are documented in the dbSNP database [37] may accelerate the search for biomedical SNP markers [41,42]. For this purpose, all the identified SNPs were mapped onto whole-genome maps of genes [38,39] and onto protein-binding sites in DNA that were predicted *in silico *[43,44] and detected *in vivo *using chromatin immunoprecipitation (ChIP), interchromosomal contacts, nucleosomes, transcriptomes either in health [45], during infection [46], disease [47], or after treatment [48]. On the basis of these data, many Web services such as TFBS [49], ACTIVITY [50], is-rSNP [51], RegulomeDB [52], rSNP-MAPPER [53], RAVEN [54], SELEX_DB [55], FunSeq2 [56], APEG [57], FeatureScan [58,59], SNPChIPTools [60], SNP-MED [61], SNAP [62], FunciSNP [63], SPOT [64], rSNP_Guide [42,65], and ChroMoS [66] facilitate the search for candidate SNP markers in terms of ranking of unannotated SNPs by their similarity to biomedical SNP markers in accordance with projections of these SNPs onto whole-genome maps. According to the Central Limit Theorem, the accuracy of this similarity-based search for candidate SNP markers should increase as the number, diversity, representativeness, and completeness of genome-wide maps increase [67].

Due to this mainstream approach, the most impressive progress has been achieved with SNPs located in protein-coding gene regions [68] because of the invariant types of disruption in both structure and function of the altered proteins regardless of the cellular conditions [69]. On the other hand, the effects of SNPs that are located in regulatory regions of genes [38,70] are still difficult to predict *in silico *[71]. Most studies in this field deal with regulatory SNPs in binding sites of TATA-binding protein (TBP) in the region [-70; -20] upstream of the transcription start of various mRNAs encoded in the human genome [72,73] (assembly of the preinitiation complex starts with binding of RNA polymerase to the anchoring TBP/DNA complex [74,75]). For this reason, model animals with a null-mutation [76] or a knockdown of TBP [77] are always inviable.

Previously, we developed a computer-assisted method for estimation of significance (Fisher's Z-score) of the difference between ancestral and minor SNP variants in terms of their effects on gene expression [78]. Later, we confirmed the predictions of this method by our independent experiments *in vitro *under both equilibrium [79] and nonequilibrium [80] conditions of the electrophoretic mobility shift assay (EMSA). Then, we verified these empirical findings using a wide range of real-time assays, such as surface plasmon resonance, stopped-flow (on a ProteOn™ XPR36 biosensor; Bio-Rad Lab., USA) [81], and fluorescence resonance energy transfer (on an SX20 spectrometer; Applied Photophysics, UK) [82]. In addition, we utilized independent results of over 100 experiments by others [83-90]. On the basis of this comprehensive validation [79-90] of the method [78], we designed the Web service SNP_TATA_Comparator (http://beehive.bionet.nsc.ru/cgi-bin/mgs/tatascan/start.pl) [91,92] for the researchers who would like to analyze certain changes in core promoters of human genes.

In the present work, we cross-validated the output of two computer-based methods: DNA sequence analysis using Web service SNP_TATA_Comparator and keyword search for articles on biochemical markers of obesity. Near the TBP-binding sites of human gene promoters, we found 22 obesity-related candidate SNP markers, including rs201381696 (obesity), rs200487063 and rs34104384 (both: obesity-caused hypertention) in the human gene *(LEP) *of leptin and rs183433761 (obesity resistance during a high-fat diet) in the human gene *(GCG) *of glucagon. We characterized them empirically and in terms of quantitative estimates of the equilibrium dissociation constant (K^0^_D_). We then validated these estimates in terms of the apparent dissociation constant, K^*^_D _≡ k_d_/k_a_, the ratio of the association rate constant (k_a_), and the dissociation rate constant (k_d_) of the TBP-DNA complex by means of EMSA under nonequilibrium conditions *in vitro*. We found a significant linear correlation (r = 0.99; α < 0.01) between K^0^_D _values predicted *in silico *and K^*^_D _values determined by measurements *in vitro *for the obesity-related candidate SNP markers rs200487063, rs201381696, rs34104384, and rs183433761, which may be useful for postgenomic predictive preventive personalized medicine.

## Results

### The results of *in silico *analysis of the known and candidate SNP markers in the TBP-binding sites of the human gene promoters

We applied our Web service SNP_TATA_Comparator [91,92] to 68 biomedical and candidate SNP markers in the TBP-binding sites of the human gene promoters taken from our recent review [92]. Table S1 (Additional file 1) shows the results.

#### The human *IL1B *gene

(interleukin 1β) promoter contains the biomedical SNP marker rs1143627. This SNP is associated with greater body fat in older men [93], Graves' disease [94], gastric cancer [95], hepatocellular carcinoma [96], non-small cell lung cancer [97], gastric ulcer and chronic gastritis [98], and major recurrent depression (99). It is a widely studied regulatory SNP marker in the TBP-binding sites of human gene promoters. Previously, we also studied this SNP *in vitro *by EMSA under both equilibrium [79] and nonequilibrium [80] conditions as well as *in silico *[83].

#### The human *APOA1 *gene

(apolipoprotein A-I) has the -35A→C substitution relative to the start of transcript number 3 of this gene. This SNP is inside a proven TATA box (the canonical form of TBP-binding sites) [10]. It is associated with obesity, fatty liver, and hematuria [10]. Using this SNP marker of obesity, we validated the suitability of our Web service SNP_TATA_Comparator for analysis of obesity-related polygenic human diseases (see Methods).

#### The human *NOS2 *gene

(inducible nitric oxide synthase 2) contains an SNP marker of epilepsy [100] and resistance to malaria [101]. It is a -51T→C substitution relative to the start of transcript number 1 [102] that causes NOS2 overexpression according to clinical research [100-102]. We have previously studied this SNP in depth using EMSA [79,80] and our computer-based method [83].

In this work, an additional keyword search (hereinafter, see Methods) pointed to new data suggesting that *NOS2 *overexpression may be a biochemical marker of obesity [103]. On the basis of this empirical observation [103], we propose the -51T→C substitution in the *NOS2 *gene promoter as a candidate SNP marker of obesity (Table S1, Additional file 1).

#### The human *PGR *gene

(progesterone receptor) contains the biomedical SNP marker rs10895068 causing *de novo *appearance of a spurious TBP-biding site along with an additional pathogenic transcription start site (TSS) at position +270 relative to the normal TSS for transcript number 2 of the same gene. This SNP is associated with endometrial cancer in obese women [104]. It was previously analyzed by our computer-based method [83].

Here, using a keyword search, we found new clinical data showing that *PGR *overexpression is a biochemical marker of male breast cancer (T4 tumor) in obesity [105]. With this in mind, we propose rs10895068 as a candidate SNP marker of breast cancer in obese men (Table S1, Additional file 1).

#### The human *ESR2 *gene

(estrogen receptor β) contains the SNP marker (rs35036378) of a primary ESR2-deficient pT1 tumor that can transform into breast cancer without preventive treatment [106]. An additional keyword search pointed to new clinical data showing that an *ESR2 *deficiency can reduce the risk of obesity after ovariectomy [107]. According to these data, we propose the SNP s10895068 as a candidate marker of the low risk of obesity after ovariectomy (Table S1, Additional file 1).

#### The human *HSD17B1 *gene

(placental 17-β-hydroxysteroid dehydrogenase) contains an SNP marker (rs201739205) of breast cancer [108]. Nevertheless, using a keyword search, we found a new promising study suggesting that weight loss in HSD17B1-deficient obese postmenopausal women by means of the appropriate diet and/or exercise reduces the risk of obesity-related cancers [109]. Thus, we propose rs201739205 as a possible predictive SNP marker of effectiveness of preventive anticancer treatment among obese postmenopausal women (Table S1, Additional file 1).

#### The human *MBL2 *gene

(soluble mannose-binding lectin 2, synonym: protein C) contains the SNP marker (rs72661131) of variable immunodeficiency [110], preeclampsia [111], and stroke [112] caused by deficient expression of this gene as we have also predicted *in silico *[83] and confirmed under both equilibrium [79] and nonequilibrium [80] conditions *in vitro*. A keyword search for "MBL2," "deficiency," and "obesity" pointed to clinical findings that an MBL2 deficiency can increase the risk of obesity [113]. Therefore, rs72661131 may be a candidate SNP marker of obesity (Table S1, Additional file 1).

Near this biomedical SNP marker, we found one more SNP: rs562962093 (unannotated), and then, predicted that it can impair the expression of MBL2, too. This is why we propose rs562962093 as a candidate SNP marker of obesity [113] (Table S1, Additional file 1).

#### The human *F7 *gene

(coagulation factor VII; synonym: proconvertin): its promoter contains a biomedical SNP, namely, the -35A→C substitution relative to the start of transcript number 1 of this gene. This SNP is a marker of moderate bleeding caused by an F7 deficiency [114].

An additional keyword search pinpointed the clinical finding of a statistically significant correlation between the total F7 level in obese patients with type 2 diabetes mellitus and the risk of cardiovascular complications [115]. Hence, we suggest the -35A→C substitution within the human *F7 *gene promoter as a candidate SNP marker of the low risk of cardiovascular complications in obese patients with type 2 diabetes mellitus (Table S1, Additional file 1).

Near this biomedical SNP marker, we found two unannotated SNPs, rs367732974 and rs549591993, that can cause overexpression of the *F7 *gene (Table S1, Additional file 1). On the basis of the correlation [115], we propose the two SNPs rs367732974 and rs549591993 as candidate markers of the high risk of cardiovascular complications in obese patients with type 2 diabetes mellitus (Table S1, Additional file 1).

#### The human *F3 *gene

(coagulation factor F3, synonym: tissue factor) contains a known SNP marker (rs563763767) of obesity [116], myocardial infarction, and thromboembolism caused by F3 overexpression [117] (Table S1, Additional file 1).

#### The human *HBB *and *HBD *genes

(β- and δ-chains of hemoglobin, respectively) contain the best-studied TBP-binding sites that are altered by a number of SNP markers of resistance to malaria and thalassemia (Cooley's anemia) [118], namely: rs34500389, rs33981098, rs33980857, rs34598529, rs33931746, rs397509430, and rs35518301. Previously, we have analyzed most of them in depth using our computer-based method [83,91] as well as using EMSA under both equilibrium [79] and nonequilibrium [80] conditions *in vitro*.

Using a keyword search, we found new clinical data showing that a hemoglobin deficiency can serve as a biochemical marker of chronic inflammation in comorbidities of obesity. This finding allows us to propose rs34500389, rs33981098, rs33980857, rs34598529, rs33931746, rs397509430, and rs35518301 as candidate SNP markers of inflammatory complications in obesity (Table S1, Additional file 1).

Near these biomedical SNP markers, we found three unannotated SNPs: rs63750953, rs281864525, and rs34166473. Our analysis predicted that they can cause a hemoglobin deficiency. Consequently, we propose them as candidate SNP markers of inflammatory complications of obesity (Table S1, Additional file 1).

After that, we analyzed all unannotated SNPs in the [-70; -20] region (where all proven TBP-binding sites are located) in the only known promoter of the **human *LEP *gene**, which we selected due to Friedman's discovery that the *LEP *gene (encodes hormone leptin) is the "obesity gene": *OB *≡ *LEP *[16] (Fig. S1, see Additional file 2). Table S1 (Additional file 1) shows three unannotated SNPs--rs201381696, rs200487063, and rs34104384--which can alter TBP's affinity for this promoter according to our prediction (see Additional file 2). The first one in the list can reduce the TBP-promoter affinity, whereas the two others can increase this affinity. Using a keyword search, we found literature data showing that the leptin deficiency is a biochemical marker of obesity [120], whereas leptin overexpression can serve as a marker of obesity-caused hypertension [6,7]. This is why we propose three more candidate SNP markers (rs201381696 as well as rs200487063 and rs34104384) of obesity-caused hypertension (Table S1, Additional file 1).

Finally, we selected one more unannotated SNP (rs183433761) within the TBP-binding site of the promoter of the **human *GCG *gene **of glucagon because this SNP's association with obesity has not yet been examined. Similarly, we predicted a *GCG *deficiency and found (using a keyword search) that it is a biochemical marker of the resistance to obesity during a high-fat diet [121]. Accordingly, we propose rs183433761 as a candidate SNP marker of obesity resistance during a high-fat diet (Table S1, Additional file 1).

We reviewed all of the above findings and selected four of our obesity-related candidate SNP markers--rs201381696, rs200487063, rs34104384, and rs183433761--for empirical biochemical verification using EMSA under nonequilibrium conditions *in vitro *because these SNPs have not yet been verified experimentally on their possible association with obesity.

### The results of *in vitro *analysis of the four selected candidate SNP markers

**Figure 1:**
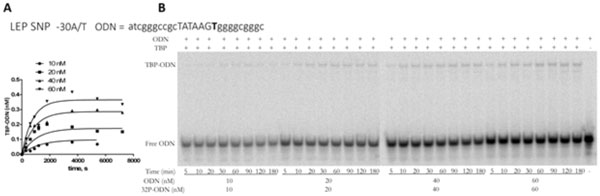
**Measurement of the kinetics of TBP binding to the oligodeoxyribonucleotide atcgggccgctataagTggggcgggc corresponding to rs34104384 (minor allele)**. A: Dependences of reaction rates on ODN concentration. B: Electropherograms from which these curves were derived. TBP concentration was 0.3 nM in all experiments; the concentrations of ODN were as indicated in the TBP/TATA-associated isotherms. The k_a _and k_d _values were calculated from the electropherograms using the GraphPad Prism 5 software (http://graphpad-prism.software.informer.com/5.01).

The primary experimental data from the *in vitro *analysis of the four selected candidate SNP markers are depicted in Figure 1. Table 1 shows these results for rs200487063, rs201381696, rs34104384, and rs183433761 in terms of the primary experimental k_a _and k_d _values that characterize the kinetics of TBP's binding to the ancestral and minor variants of the TBP-binding site in the only promoter of the *LEP *gene that is documented in the human reference genome hg19.

**Table 1:** EMSA-based analysis of the complex of TBP and one of oligodeoxyribonucleotides (ODNs) *in vitro*.

Gene probe	^32^P-labeled synthetic ODN, 26 bp, double-stranded DNA	k_a_, × 10^3 ^M^-1^s^-1^	k_d_, × 10^-4 ^s^-1^	K^*^_D_, nM	t_1/2_, min	-ΔG Kcal/mol
*LEP *WT	atcgggccgcTATAAgaggggcgggc	2.3 ± 0.6	1.8 ± 0.6	78	64 ± 13	9.7 ± 0.9
*LEP *-38**a**	atcgggcc**a**cTATAAGaggggcgggc	3.5 ± 0.5	1.5 ± 0.2	43	77 ± 16	10.0 ± 1.0
*LEP *-30**t**	atcgggccgcTATAAG**t**ggggcgggc	11.0 ± 2.0	8.0 ± 1.0	73	14 ± 3	9.7 ± 1.0
*LEP *-35**g**	atcgggccgcT**g**TAAGaggggcgggc	5.6 ± 0.8	13.0 ± 2	232	9 ± 1	9.0 ± 0.9

*GCG *WT	agctggagagTATATAaaagcagtgc	70 ± 10	5 ± 1.0	8	23 ± 5	11.1 ± 1.0
*GCG *-41**g**	agctggagagT**g**TATAaaagcagtgc	30 ± 10	6 ± 1.0	18	19 ± 4	10.5 ± 0.9

**Notes: **For each TBP-ODN-complex, k_a _is the association rate constant, k_d _is the dissociation rate constant, half-life (t_1/2_) equals (ln2)/k_d_, and K^*^_D _= k_d_/k_a _is the apparent dissociation constant. TATA-like subsequence: uppercase letters; ΔG = -RTln(_ka_/k_d_) is a change in Gibbs free energy, where R = 1.38 × 10^-23 ^JK^-1 ^is Boltzmann's constant, T is temperature in degrees Kelvin; the substitution corresponding to the minor allele of each SNP is shown in boldface.

First, the above-mentioned results show that the variant -38a in the *LEP *gene promoter increases the rate of formation of the TBP-DNA complexes (k_a_) by 50%, the variant -30t fivefold, and variant -35g by 24-fold. The rate of decay (k_d_) for -38a increased by 20%, and for -35g by 14-fold, whereas for -30t, it decreased 4.4-fold. Affinity (K^*^_D_) of TBP for ODN containing -38a increased by 50%, and for the -30t variant it increased by 12%, whereas for the -35g variant, the affinity diminished 2.8-fold. Meanwhile, the minimal half-life of the complex (t_1/2_) of 9 min was observed for the -35g variant; this situation was caused by the increase in the rate of day (k_d_) 14-fold relative to the norm and by very low affinity. It should be noted that the variants -38a and -30t are located in the flanks of the TATAbox (the canonical form of a TBP-binding site), and only variant -35g affects the sequence of the TATAbox itself, thereby causing a massive decrease in affinity to 230 nM.

As shown in Table 1 the greatest increase in the rate of formation and decay of the TBP-TATA complex was observed for variant -35g (24-fold and 14-fold, respectively), which affects the sequence of the TATA-box and replaces its most conserved base "A" with "G" [122]. This change reduces affinity (K^*^_D_) to a virtually nonspecific level (230 nM) and reduces half-life of the complexes more than sevenfold. This alteration is associated with obesity [120].

In contrast, variant -41g of the promoter of *GCG *lowers the rate of formation (k_a_) of the complex TBP-ODN 2.3-fold, whereas the rate of dissociation (k_d_) of the complex and its half-life decrease by 20%. The calculated K_D _value, characterizing the change in TBP-ODN affinity, decreased 2.25-fold.

At the same time, their ratio, k_d_/k_a_, which determines the apparent dissociation constant K^*^_D _and affinity TBP-ODN, decreased more than threefold in the case of -35g, which disrupts the TATA-like subsequence (by converting it to g_-38_cTg_-35_TAA) but increased 1.6-fold for -38a flanking the normal a_-38_cTA_-35_TAA pentanucleotide. We estimated the half-life of the TBP-ODN complexes (Table 1). Its values, 9-77 min, fit the time interval, 5-180 min (Figure 1), of nonequilibrium *in vitro *conditions for our measurement of the kinetics of TBP's binding to each ODN corresponding to our three possible obesity-related SNP markers. The minimal half-life, 9 min, was observed for the allele -35G which corresponds to minimal binding affinity and to a maximal dissociation rate of the complex TBP-TATA.

Thus, judging by the effects of rs200487063, rs201381696, rs34104384, and rs183433761, the constants k_a _and k_d _describe qualitatively different and independent characteristics. This observation is in agreement with the commonly accepted notion that K^*^_D_, k_a_, and k_d _independently characterize different features of the kinetics of intermolecular binding.

**Figure 2:**
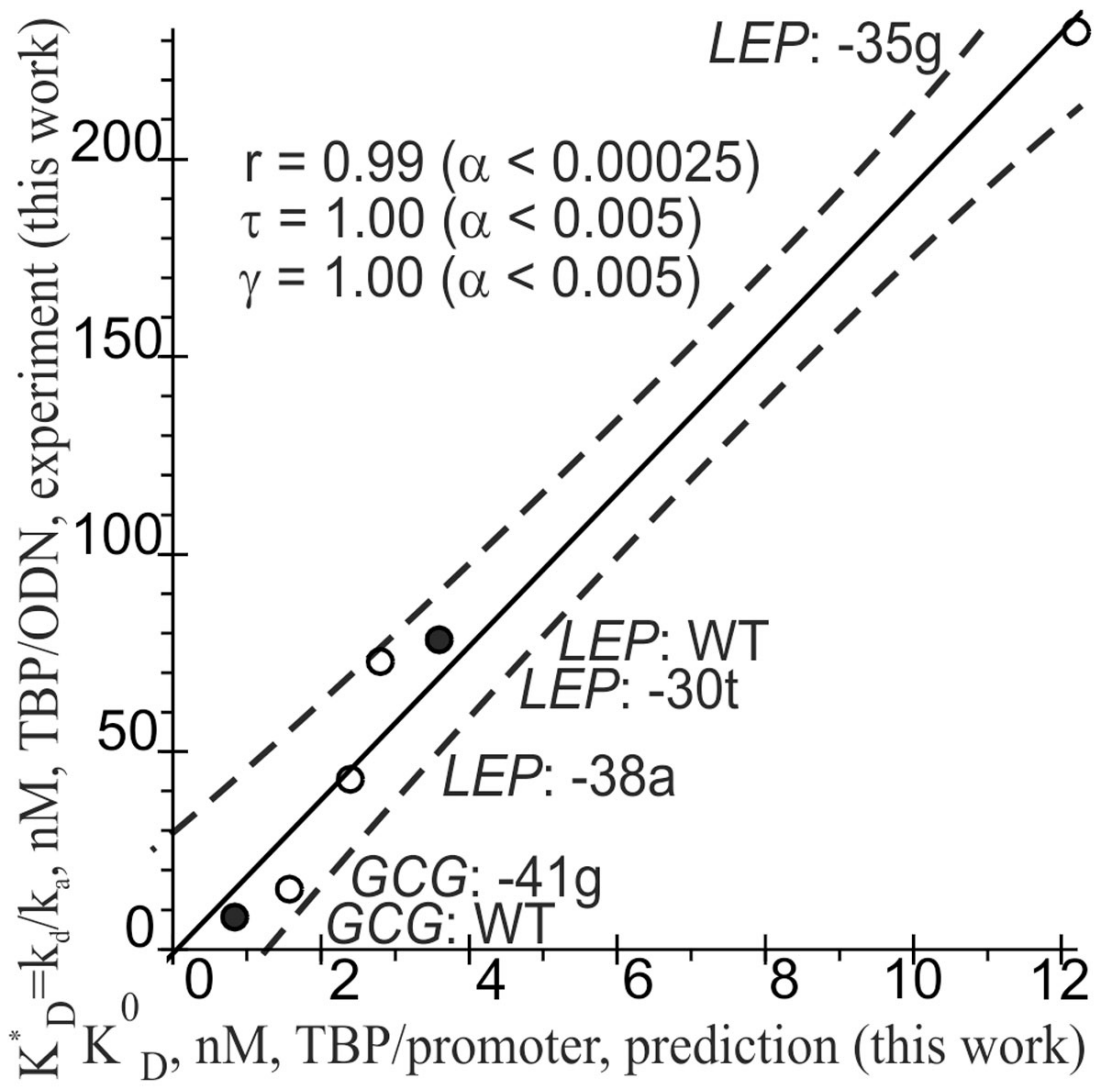
The significant correlations between the predicted K^0^_D _values and K^*^_D _values determined by measurement *in vitro*. *Legend: *Solid and dashed lines denote the linear regression and boundaries of its 95% confidence interval, calculated by the software package STATISTICA (Statsoft™, USA); ● and ○ are the ancestral (hg19) and minor alleles, respectively, of the four possible obesity-related SNP markers within the human *LEP *and *GCG *gene promoters; r, τ, γ, and α are coefficients of the Pearson's simple linear correlation, Kendall's rank correlation, and Goodman-Kruskal's generalized correlation and their significance, respectively.

Figure 2 shows significant correlations between nonequilibrium *in vitro *measurements of K^*^_D _(Table 1) and equilibrium *in silico *estimates of K^0^_D _(Table S1, see Additional file 1). Assessment by three independent statistical tests is provided: Pearson's simple linear correlation (r = 0.99; α < 0.00025), Kendall's rank correlation (τ = 1; α < 0.005), and Goodman-Kruskal's generalized correlation (γ = 1; α < 0.005). These data are indicative of robustness of the correlation, and this robustness is consistent with our analyses of various SNP markers of monogenic diseases under equilibrium [79], nonequilibrium [80], and real-time [82] experimental *in vitro *conditions and with independent studies by other authors [74,123,124].

The different ranges of K^0^_D _and K^*^_D _reflect the differences in uncontrollable factors under equilibrium and nonequilibrium *in vitro *conditions, which do not influence the relative values of TBP affinity for different variants of the TBP-binding site (Figure 2). Because of this property of TBP-binding sites (Figure 2), which was proven empirically by many research groups [74,123,124], leptin production in a patient with the minor variant -35g of rs201381696 may be proportionately lower (in each tissue expressing the *LEP *gene, regardless of its tissue specificity) in comparison with a person carrying the variant WT. Consequently, the total plasma leptin level in this patient will be lowered: this is a clinically established risk factor of obesity [120]. By the same token, the minor variants -38g of rs200487063 and -30t of rs34104384 may cause a proportional increase in leptin production (by any tissues) and a proportional increase in the overall plasma leptin level, which is a known risk factor of hypertension as a complication of obesity [6,7]. This combined empirical and computer-based identification of rs200487063, rs201381696, rs34104384, and rs183433761 (never considered in association with obesity) as obesity-related candidate SNP markers is the main result of this work.

## Discussion

Here, we focused only on the TBP-binding site because it is the best-studied site in human gene promoters [125]; we detected known SNP manifestations: "susceptibility/resistance" to diseases. In comparison with our previous applications of the Web service SNP_TATA_Comparator [91,92] to the research on monogenic diseases [78-92], here we found a number of different associations, such as complications of obesity (e.g., rs10895068, male breast cancer [T4 tumor] in obesity), a phenotypic trait (e.g., rs1143627, greater body fat in older men), consequences of surgery (e.g., rs35036378, reduced risk of obesity after ovariectomy), eating behavior (e.g., rs183433761, obesity resistance during a high-fat diet), and the preventive effect of a lifestyle (e.g., rs201739205, low risk of obesity-related cancers due to weight loss by diet/exercise in obese postmenopausal women). These findings can extend the practical utility of our Web service because of the cross-validation of its output with a keyword search for available literature on an appropriate biochemical marker of the diseases under study.

In addition to the commonly used textual analysis of DNA sequences, we take into account quantitative values of the minor-groove width of B-helical DNA [125] because TBP binds to DNA via incorporation of side chains of two amino acid residues into DNA's minor groove [126]. This additional criterion increases the accuracy of our prediction (Figure 2), in agreement with independent findings of some other researchers [127]. This result means that the computer-based SNP analysis may be improved if various quantitative parameters of conformational and/or physicochemical features of B-helical DNA [128,129] are taken into account in addition to the textual data.

Furthermore, population frequencies of the minor alleles of unannotated SNP can serve as another information source that may improve the predictions of candidate SNP markers of a disease. Accordingly, Table S1 (Additional file 1) characterizes all the SNPs under study in terms of the population frequencies taken from the "1000 Genome Browser" [131]. As one can see, these parameters vary from sample to sample in a wide range (e.g., the population frequency rates of rs1143627 vary from 27% to 65%, see Table S1, Additional file 1). In addition, many biomedical SNP markers still had no values of population frequencies within the framework of the "1000 Genome Browser" [131] (e.g., rs397509430, rs33980857, rs34598529, rs33931746, rs33981098, rs34500389, rs63750953, rs281864525, rs35518301, and rs34166473; see Table S1, Additional file 1). Moreover, many biomedical SNP markers were not still documented by the database dbSNP [37] (e.g., the substitutions -35A→C (*APOA1)*, -51T→C (*NOS2*), -35A→C (*F7*); see Table S1, Additional file 1). At present, the above-mentioned population frequency values correspond to ethnic groups in regional subpopulations (for studies on human migration flows) rather than cohorts of patients with certain risk factors of diseases--overweight, smoking, and alcohol intake--to prioritize the candidate SNP markers according to the biomedical standards [109]. This is why the type of biomedical standardization of whole-genome data available today may advance postgenomic predictive preventive personalized medicine [30].

Our *in vitro *assays (Table 1) and *in silico *predictions (Table S1, Additional file 1) all indicate that the greatest changes in the leptin level may be expected for the minor allele -35g, which disrupts the TATA-like TBP-binding site and decreases the TBP-DNA affinity threefold. In contrast, the minor alleles -30t and -38a flanking this site seem to enhance this affinity by 25% and 50%, respectively. We have already observed this pattern empirically in a previous study, namely, three SNP markers of β-thalassemia (rs34598529, rs33931746, and rs3393174) within the well-known TATA box decrease TBP's affinity for the human *HBB *gene promoter 10-fold, whereas another biomedical SNP marker, -27A→t (flanking this TATA box), yields only a 20% decrease [80]. As for the smallest change in the binding affinity of TBP for the promoter variant -30t (rs34104384, *LEP*), one can see in Table S1 (Additional file 1) that the same or even smaller effects are observed for some biomedical SNP markers, such as rs35036378 (25%, *ESR2*), the -35A→c substitution (25%, *APOA1*), rs34500389 (17%, *HBB*), and the -35A→c substitution (15%, *F7*). In addition, one can see in Table 1 that the quantitative values of the Gibbs free energy change (ΔG) are an unreliable criterion for distinguishing between variants of ODNs binding to TBP. In contrast, the kinetic constant k_a _of the association rate of the TBP-ODN complex does it best (see Results). This finding means that the binding of TBP to a promoter is under kinetic control rather than under thermostatic one. Therefore, additional empirical measurements can enrich the preliminary bioinformatics predictions on the candidate SNP markers of diseases.

Finally, our combined *in silico *and *in vitro *data may serve as a good rationale for clinical researchers who wish to validate promising candidates for SNP markers. The definitive proof of an SNP as a clinical marker is demonstration of a significant difference in its frequency between patients and healthy people, with adjustments for various confounding factors such as the ethnic and gender composition of the regional subpopulation in question, lifestyle, living conditions, climate, environment, and expressivity and penetrance of the disorder under study [71].

## Conclusions

Changes in the affinity of a transcription factor for a regulatory DNA sequence are commonly an appreciable but not a crucial factor of a disease; for this reason, diverse clinical manifestations of the interactions between genetic and environmental factors are typical of polygenic diseases [132]. In the case of metabolic syndrome [1], obesity is a strong contributing factor [2] in addition to genetic predisposition. Prevalence of obesity among overweight Europeans exceeds 50% and results in elevated risk of respiratory failure, atherosclerosis, and heart failure [133]. In this work, we identified 22 obesity-related candidate SNP markers. Their validation in accordance with proper biomedical standards may help to solve the global problem of treatment of metabolic syndrome [1] by means of the postgenomic predictive preventive personalized medicine [30].

## Methods

### *In vitro *analysis

Recombinant full-length human TBP (native amino acid sequence) was expressed in *Escherichia coli *BL21 (DE3) cells transformed with the pAR3038-TBP plasmid (the generous gift of Prof. B. Pugh, Pennsylvania State University) by a previously described method [134] with two modifications: the IPTG concentration was 1.0 instead of 0.1 mM, and the induction time was 3 instead of 1.5 h. For a more detailed description of our protocol for production and purification of human TBP, see [79].

Oligodeoxyribonucleotides (ODNs) 26 bp long were synthesized by the Biosynthesis Enterprise (Novosibirsk, Russia) and were purified by PAGE. The ODN sequences correspond to either the ancestral or minor allele of SNPs of the TBP-binding site in the promoters of human genes *LEP *and *GCG *that are analyzed here *in vitro *(Table 1). Labeled double-stranded ODNs were prepared by ^32^P labeling of both strands by means of T4 polynucleotide kinase (SibEnzyme, Novosibirsk) with subsequent annealing by heating to 95°C (at equimolar concentrations) and slow cooling (no less than 3 h) to room temperature. The duplexes were analyzed in 15% nondenaturing polyacrylamide gel (1 × Tris-borate-EDTA buffer) and isolated by electroelution. For a detailed description of our protocol for labeling of ODNs with ^32^P, see [79].

The association rate constant (k_a_) and dissociation rate constant (k_d_) were determined for the complexes of TBP with each 26-bp ODN corresponding to the 26-bp sequence of either the ancestral or minor variant of the human *LEP *gene promoter. Association kinetics experiments were performed at four ODN concentrations: 10, 20, 40, and 60 nM as shown in Figure 1(B). The experiments with TBP/ODN binding were performed at 25°C in binding buffer (20 mM 4-[2-hydroxyethyl]-1-piperazineethanesulfonic acid [HEPES]-KOH pH 7.6, 5 mM MgCl_2_, 70 mM KCl, 1 mM dithiothreitol [DTT], 100 μg/mL BSA, 0.01% NP-40, and 5% glycerol) at a fixed concentration (0.3 nM) of active TBP. The gels were dried and Imaging Screen-K (Kodak, Rochester, NY, USA) was exposed to these gels for analysis on a Molecular Imager PharosFX Plus phosphorimager (Bio-Rad, Herts, UK). The resulting autoradiographs were quantitated in the Quantity One 4.5.0 software (Bio-Rad) as shown in Figure 1(A). Using these data, we calculated the association rate constant (k_a_) and dissociation rate constant (k_d_) in the Graph-Pad Prism 5 software (http://graphpad-prism.software.informer.com/5.01) by global fitting of the data onto the association kinetics model. For a detailed description of our protocol for *in vitro *measurement of association and dissociation rate constants for TBP/ODN complexes, see [80].

### *In silico *analysis

We analyzed DNA sequences of the human gene promoters taken from the database Ensembl [38] using their locations within the human reference genome (hg19) taken from the database GENCODE [135], as shown in Fig. S2 (Additional file 3). For each DNA sequence, we assessed the maximal "-ln(K^0^_D_) ± δ" affinity of TBP for the [-70; -20] promoter region (where all the known sites are located) using our Web service [91,92] as described in Additional file 4. For each case of predicted significant overexpression or downregulation of the human genes (as clinically relevant biochemical markers), we manually performed a keyword search in the NCBI databases [136] as described in detail elsewhere [137] and schematically shown in Fig. S3 (Additional file 5). Our heuristic interpretation of these predicted cases of significant overexpression and underexpression of the human genes is shown in the second rightmost column of Table S1 (Additional file 1): these are clinical data identified by our manual keyword search, with citations in the rightmost column.

Finally, using the software package STATISTICA (Statsoft™, Tulsa, USA), we estimated statistical significance of three correlations: Pearson's simple linear correlation (r), Kendall's rank correlation (τ), and Goodman-Kruskal's generalized (γ) correlation between the K^0^_D _values predicted *in silico *and the K^*^_D _values obtained by measurement *in vitro *as shown in Figure 2.

## Abbreviations

ApoA1, apolipoprotein A1; EMSA, electrophoretic mobility shift assay; GCG, glucagon; HBB, hemoglobin subunit β; HBD, hemoglobin subunit δ; k_a_, association rate constant; k_d_, dissociation rate constant; K^*^_D_, apparent dissociation constant; LEP, leptin; ln, natural logarithm; ODN, oligodeoxyribonucleotide; SNP, single nucleotide polymorphism; t_1/2_, half-life; TBP, TATA-binding protein; TSS, transcription start site; WT, wild type (norm); ΔG, Gibbs free energy change.

## Competing interests

The authors declare that they have no competing interests.

## Authors' contributions

MPP performed the sequence analysis and helped to draft the manuscript. OVA, IAD, and TVA carried out the *in vitro *experiments. DAR designed, developed, maintained, adapted, and tuned the software for both sequence and data analysis. PMP designed and performed the statistical analysis. LKS drafted the manuscript and designed and coordinated the *in vitro *experiments. NAK conceived of and supervised the study. All coauthors read and approved the final version of the manuscript.

## Supplementary Material

Additional file 1 **Table S1**. Obesity-related known and candidate SNP markers altering affinity of the TATA-binding protein (TBP) for human gene promoters. Click here for file

Additional file 2 **Figure S1**. The obesity-related candidate SNP markers (in the human LEP gene promoter) predicted using SNP_TATA_Comparator [92]. (A) The only promoter of the human *LEP *gene and the six unannotated SNPs (analyzed in this study) in the region [-70; -20] (double-headed arrow, ↔) where all proven TBP-binding sites are located. Single-headed arrow (→): transcription start site (TSS), box: TATA-like subsequence TATAA, ellipse: three possible obesity-related SNP markers predicted in this work. (B-D) The results produced by our Web service SNP_TATA_Comparator [92] for the three possible obesity-related SNP markers (rs200487063, rs201381696, and rs34104384) located in the human *LEP *gene promoter. The symbols are explained in the legend of Fig. S2 (see Methods; Additional file 3). Click here for file

Additional file 3 **Figure S2**. The result produced by SNP_TATA_Comparator [92] for a known SNP marker of obesity [10]. *Legend: *Solid, dotted, and dashed arrows indicate queries for the gene list, list of transcripts of a certain gene, and DNA sequence of the promoter corresponding to the specified transcript of the gene in Ensembl [38] and GENCODE [135] editions of the reference human genome hg19, respectively. Dash-and-dot arrows: estimates of significance of the aberration of gene product abundance in patients with the minor allele (relative to the ancestral allele: reference human genome hg19) expressed as Fisher's Z-score. Two circles indicate the ancestral allele (-35A) and minor allele (-35c) of this SNP marker of obesity (-35A→c); this SNP causes underexpression the human *APOA1 *gene [10]. Click here for file

Additional file 4 **Supplementary method**. A quantitative estimate of binding affinity of TATA-binding protein (TBP) for a promoter of the human gene as a function of DNA sequence of this promoter. Click here for file

Additional file 5 **Figure S3**. A flow chart of the keyword search for comorbidities of obesity where biochemical markers correspond to a change in expression of a given gene containing the SNP marker of interest. Click here for file

## Declarations

Publication of this article has been funded by the Russian Science Foundation.

This article has been published as part of *BMC Genomics *Volume 16 Supplement 13, 2015: Selected articles from the 7th International Young Scientists School "Systems Biology and Bioinformatics" (SBB'2015): Genomics. The full contents of the supplement are available online at http://www.biomedcentral.com/bmcgenomics/supplements/16/S13.
