# Supplementary material for: Obesity-related known and candidate SNP markers can significantly change affinity of TATA-binding protein for human gene promoters
Source: BMC Genomics. 2015 Dec 16;16(Suppl 13):S5. doi: 10.1186/1471-2164-16-S13-S5 (PMC4686794; doi:10.1186/1471-2164-16-S13-S5)
Supplement: Additional file 1 — Table S1. Obesity-related known and candidate SNP markers altering affinity of the TATA-binding protein (TBP) for human gene promoters. [file 1471-2164-16-S13-S5-S1.pdf]

Additional file 1: Table S1: Obesity-related known and candidate SNP markers altering affinity of the TATA-binding protein (TBP) for human gene promoters

Gene	dbSNP [37] rel. 142 or see [Reference]	p, %, minor allele		5′ flank	**hg19** / *min*	3′ flank	K_D, nM		Z-score		known diseases (observations) [Reference] *or hypothetical ones in the case of the candidate SNP markers predicted by us in [this work] (see Methods: Fig. S3, Additional file 5)*	[Reference] or *[this work]*
		mean	{range} (max: sample)				**hg19** / *min*	Δ	Z	α		
IL1B	rs1143627	47	{27 - 65} (CEU)	ttttgaaagc	**c** / *t*	ataaaaacag	**5** / *2*	↑	15	10^{-7}	greater body fat in older men; also gastric cancer, hepatocellular carcinoma, non-small cell lung cancer, chronic gastritis and gastric ulcer, Graves' disease, major recurrent depression	[93-99]
APOA1	ND, see [107]			tgcagacata	**a** / *c*	ataggccctg	**3** / *4*	↓	5	10^{-3}	obesity; also hematuria, fatty liver	[10]
NOS2	ND, see [102]			gtataaatac	**t** / *c*	tcttggctgc	**2** / *1*	↑	3	10^{-2}	resistance to malaria, epilepsy risk, *also (hypothetically) obesity*	[100-102] *[this work] [103]*
PGR	rs10895068	1	{0 - 7} (CEU)	gggagataaa	**g** / *a*	gagccgcgtg	**10** / *6*	↑	8	10^{-7}	endometrial cancer in obese women [*de novo* occurrence of a spurious pathogenic TBP-biding site], *also (hypothetically) male breast cancer (T4 tumor) in obesity*	[104], *[this work] [105]*
ESR2	rs35036378	1	{0 - 7} (LWK)	cctctcggtc	**t** / *g*	ttaaaaggaa	**6** / *8*	↓	5	10^{-3}	ESR2-deficient pT1 tumor, *also (hypothetically) reduced risk of obesity after ovariectomy*	[106] *[this work] [107]*
HSD17B1	rs201739205	1	{0 - 2} (CLM)	aggtgatatc	**a** / *c*	agcccagagc	**13** / *18*	↓	5	10^{-3}	breast cancer, *also (hypothetically) low risk of obesity-related cancers due to weight loss by diet/exercise in obese postmenopausal women*	[108] *[this work] [109]*
MBL2	rs72661131	0.1	{0 - 1} (MSL)	tctatttcta	**t** / *c*	atagcctgca	**2** / *4*	↓	12	10^{-7}	variable immunodeficiency, stroke, preeclampsia, *also (hypothetically) obesity*	[110-112] *[this work] [113]*
	rs562962093	0.02	1 (MSL)	atctatttct	**a** / *g*	tatagcctgc	**2** / *5*	↓	15	10^{-7}	*(hypothetically) obesity; also stroke, variable immunodeficiency, preeclampsia*	*[this work]*
F7	ND, see [114]			ccttggaggc	**a** / *c*	gagaactttg	**53** / *62*	↓	3	10^{-2}	moderate bleeding, *also (hypothetically) low risk of cardiovascular complications in obese patients with type 2 diabetes mellitus*	[114], *[this work], [115]*
	rs367732974			aactttgccc	**g** / *a*	tcagtcccat	**53** / *47*	↑	*2*	*0.05*	*(hypothetically) high risk of cardiovascular complications in obese patients with type 2 diabetes mellitus*	*[this work]*
	rs549591993	0,06	{0 - 1} (IBS)	gcccgtcagt	**c** / *a*	ccatggggaa	**53** / *25*	↑	*13*	*10^{-7}*		

Notes. Sample: the acronyms used in the window "Populations/Samples" of the "1000 Genomes Browser" [131], namely: CEU is Utah Residents, LWK is Luhya in Webuye (Kenya), CLM is Colombians from Medellin (Columbia), MSL is Mende in Sierra Leone, IBS is the Iberian population in Spain, PUR is Puerto Ricans from Puerto Rico; hg19, ancestral allele; min, minor allele; K_D, an estimate [92] of the dissociation constant (K_D) of the TBP-DNA complex *in vitro* [78]; ND, not documented; Δ, the expression change in comparison with the norm: overexpression (↑), deficient expression (↓); Z, Z-score; α = 1 – p, significance, where p is probability; Fig. S1 (Additional file 2) and S2 (Additional file 3).

Table S1: (continued)

Gene	dbSNP [37] rel. 142 or see [Reference]	p, %, minor allele: mean	p, %, minor allele: {range} (max: sample)	5′ flank	**hg19** / *min*	3′ flank	K_D, nM: **hg19** / *min*	Δ	Z-score: Z	Z-score: α	known diseases (observations) [Reference] *or hypothetical ones in the case of the candidate SNP markers predicted by us in [this work] (see Methods: Fig. S3, Additional file 5)*	[Reference] or *[this work]*
F3	rs563763767	0.08	{0 - 1} (MSL)	ccctttatag	**c** / *t*	gcgcggggca	**3** / *2*	↑	6	10^{-7}	obesity, also myocardial infarction and venous thromboembolism	[116] [117]
HBB	rs397509430			gggctgggca	**t** / *–*	atacaacagt	**5** / *29*	↓	34	10^{-7}	malaria resistance, β-thalassemia, *also (hypothetically) chronic inflammation in comorbidities of obesity*	[118] *[this work]* *[119]*
	rs33980857			gggctgggca	**t** / *a,g,c*	atacaacagt	**5** / *21*	↓	27	10^{-7}		
	rs34598529			ggctgggcat	**a** / *g*	aaagtcaggg	**5** / *18*	↓	24	10^{-7}		
	rs33931746			gctgggcata	**a** / *g,c*	aagtcagggc	**5** / *11*	↓	14	10^{-7}		
	rs33981098			agggctgggc	**a** / *g,c*	taaaagtcag	**5** / *9*	↓	10	10^{-7}		
	rs34500389			cagggctggg	**c** / *a,t,g*	ataaaagtca	**5** / *6*	↓	3	10^{-2}		
	rs63750953			*ctgggcataa*	**aa** / *–*	*gtcagggcag*	**5** / *8*	↓	*9*	*10^{-7}*	*(hypothetically) chronic inflammation in comorbidities of obesity; also malaria resistance, β-thalassemia*	*[this work]*
	rs281864525			*tgggcataaa*	**a** / *c*	*gtcagggcag*	**5** / *7*	↓	*7*	*10^{-7}*		
HBD	rs35518301			caggaccagc	**a** / *g*	taaaaggcag	**4** / *8*	↓	11	10^{-7}	malaria resistance, δ-thalassemia, *also (hypothetically) chronic inflammation in comorbidities of obesity*	[118] *[this work]* *[119]*
	rs34166473			aggaccagca	**t** / *c*	aaaaggcagg	**4** / *8*	↓	*18*	*10^{-7}*	*(hypothetically) chronic inflammation in comorbidities of obesity; also malaria resistance, δ-thalassemia*	*[this work]*
LEP	rs201381696			tcgggccgct	**a** / *g*	taagaggggc	**4** / *12*	↓	17	10^{-7}	*(hypothetically) obesity*	*[this work]*, *[120]*
	rs200487063			tgatcgggcc	**g** / *a*	ctataagagg	**4** / *2*	↑	6	10^{-7}	*(hypothetically) obesity-caused hypertension*	*[this work]* *[6, 7]*
	rs34104384	1	{0 - 7} (LWK)	ccgctataag	**a** / *t*	ggggcgggca	**4** / *3*	↑	4	10^{-2}		
GCG	rs183433761	0.04	{0 - 1} (PUR)	gctggagagt	**a** / *g*	tataaaagca	**1** / *2*	↓	17	10^{-7}	*(hypothetically) obesity resistance during a high-fat diet*	*[this work]*, *[121]*
