# Supplementary figures and images for: Obesity-related known and candidate SNP markers can significantly change affinity of TATA-binding protein for human gene promoters
Source: BMC Genomics. 2015 Dec 16;16(Suppl 13):S5. doi: 10.1186/1471-2164-16-S13-S5 (PMC4686794; doi:10.1186/1471-2164-16-S13-S5)

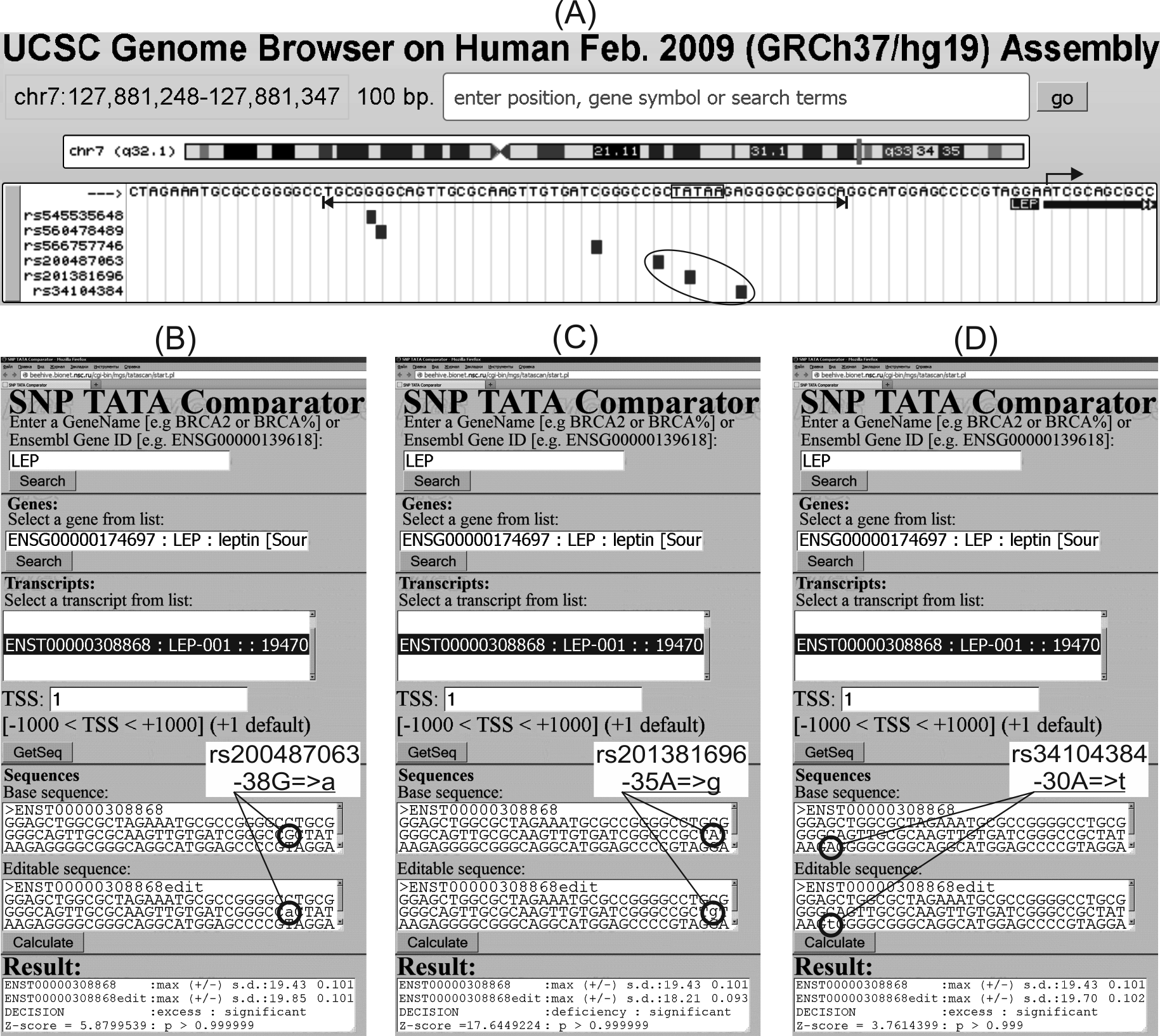

Supplement: Additional file 2 — Figure S1. The obesity-related candidate SNP markers (in the human LEP gene promoter) predicted using SNP_TATA_Comparator [92]. (A) The only promoter of the human LEP gene and the six unannotated SNPs (analyzed in this study) in the region [-70; -20] (double-headed arrow, ↔) where all proven TBP-binding sites are located. Single-headed arrow (→): transcription start site (TSS), box: TATA-like subsequence TATAA, ellipse: three possible obesity-related SNP markers predicted in this work. (B-D) The results produced by our Web service SNP_TATA_Comparator [92] for the three possible obesity-related SNP markers (rs200487063, rs201381696, and rs34104384) located in the human LEP gene promoter. The symbols are explained in the legend of Fig. S2 (see Methods; Additional file 3). [file 1471-2164-16-S13-S5-S2.png]

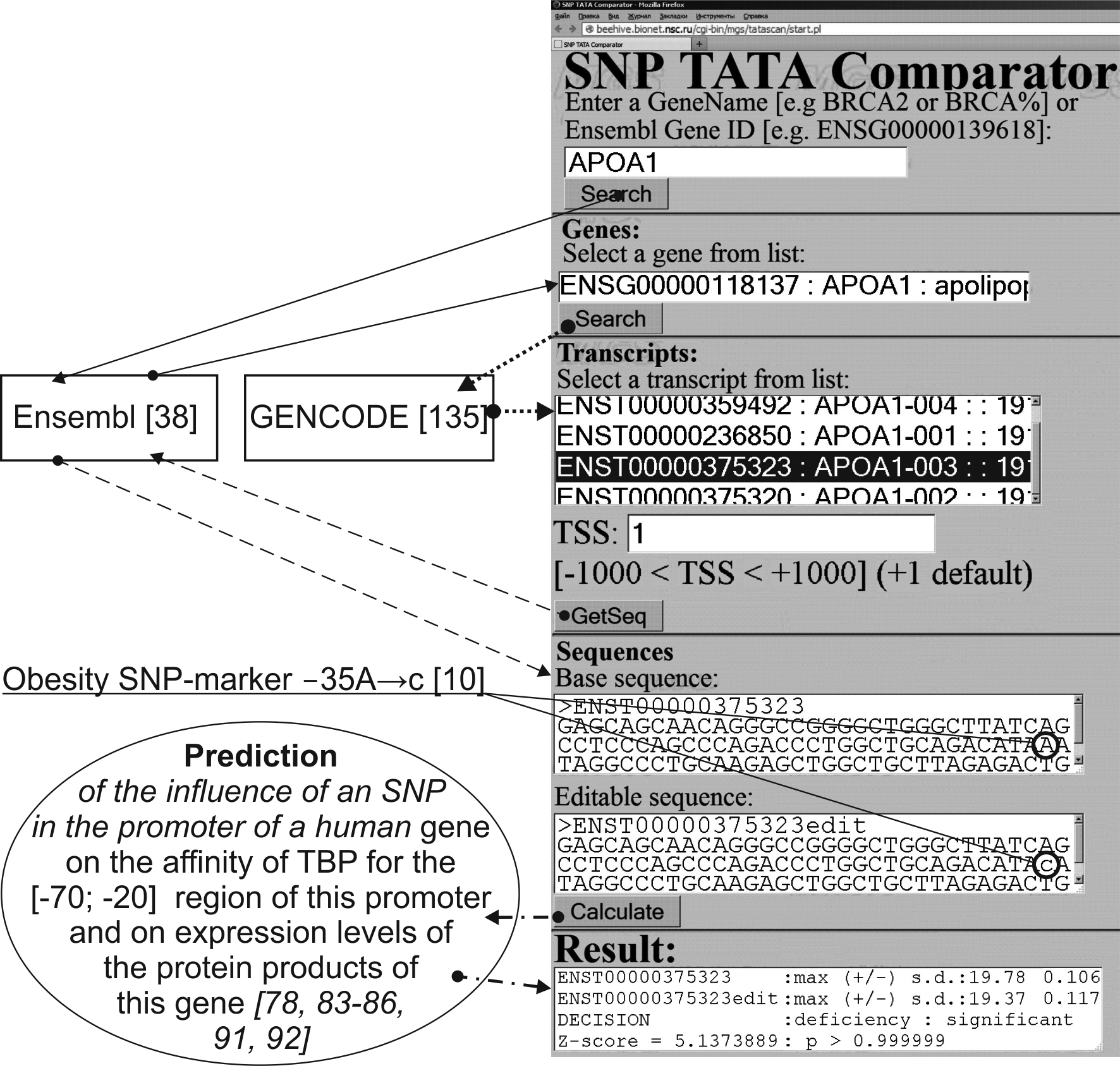

Supplement: Additional file 3 — Figure S2. The result produced by SNP_TATA_Comparator [92] for a known SNP marker of obesity [10]. Legend: Solid, dotted, and dashed arrows indicate queries for the gene list, list of transcripts of a certain gene, and DNA sequence of the promoter corresponding to the specified transcript of the gene in Ensembl [38] and GENCODE [135] editions of the reference human genome hg19, respectively. Dash-and-dot arrows: estimates of significance of the aberration of gene product abundance in patients with the minor allele (relative to the ancestral allele: reference human genome hg19) expressed as Fisher's Z-score. Two circles indicate the ancestral allele (-35A) and minor allele (-35c) of this SNP marker of obesity (-35A→c); this SNP causes underexpression the human APOA1 gene [10]. [file 1471-2164-16-S13-S5-S3.png]

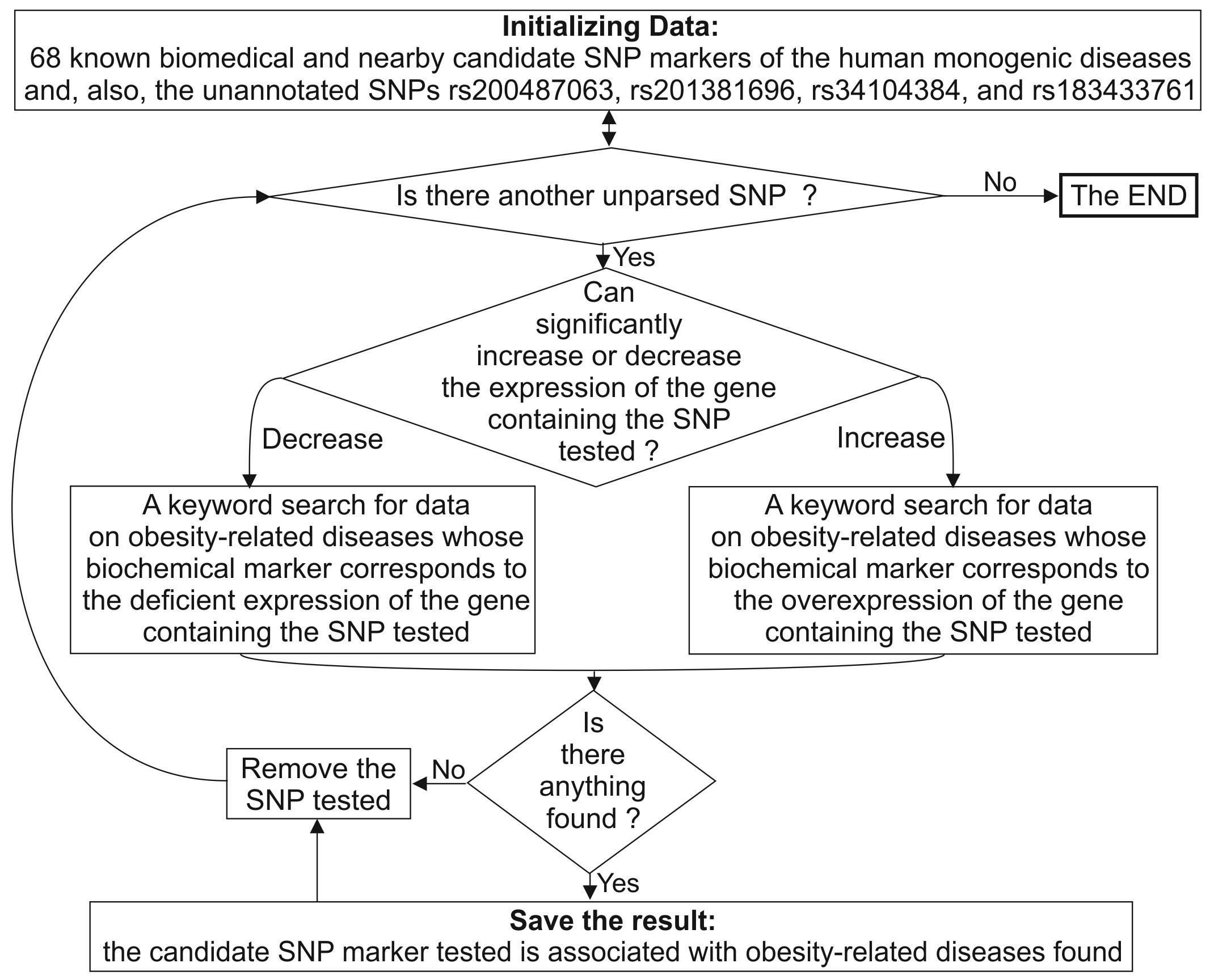

Supplement: Additional file 5 — Figure S3. A flow chart of the keyword search for comorbidities of obesity where biochemical markers correspond to a change in expression of a given gene containing the SNP marker of interest. [file 1471-2164-16-S13-S5-S5.png]
