# Supplementary material for: Obesity-related known and candidate SNP markers can significantly change affinity of TATA-binding protein for human gene promoters
Source: BMC Genomics. 2015 Dec 16;16(Suppl 13):S5. doi: 10.1186/1471-2164-16-S13-S5 (PMC4686794; doi:10.1186/1471-2164-16-S13-S5)
Supplement: Additional file 4 — Supplementary method. A quantitative estimate of binding affinity of TATA-binding protein (TBP) for a promoter of the human gene as a function of DNA sequence of this promoter. [file 1471-2164-16-S13-S5-S4.pdf]

Additional file 4: Supplementary Method**.**

A quantitative estimate of binding affinity of TATA-binding protein (TBP) for a promoter of the human gene as a function of DNA sequence of this promoter.

The input data consisted of the 90-bp DNA sequence $\{s_{-90}\ldots s_i\ldots s_{-1}\}$ in the proximal promoter region immediately upstream of the transcription start site (TSS, s_0) (where $s_i \in \{a, c, g, t\}$).

The output data included the maximal value, $-\ln(K_D) \pm \delta$, among all the possible estimates of TBP's binding affinity for the 26-bp DNA fragment, $\{s_{i-13}\ldots s_i\ldots s_{i+12}\}$ at the i-th position ranging from –70 to –20 for both DNA chains (where, K_D is the equilibrium dissociation constant expressed in moles per liter; M).

We used the three-step approximation of TBP's binding to the [–70; –20] region of the eukaryotic gene promoters: (i) TBP slides along DNA ↔ (ii) the sliding TBP stops at a proper TBP-binding site ↔ the DNA helix bends to the 90° angle and stabilizes the local TBP-promoter complex. This binding is estimated empirically as follows:

$$-\ln(K_D) = 10.9 - 0.2\ \{\ln(K_{SLIDE}) + \ln(K_{STOP}) + \ln(K_{BEND})\}, \qquad \textbf{(1)}$$

where 10.9 (ln units) is nonspecific TBP-DNA affinity (10^{-5} M), 0.2 is the stoichiometric coefficient, and K_{STOP} is the equilibrium constant of the second step of the TBP stops at a TBP-binding site (the maximal score value of Bucher's position-weight matrix, the commonly accepted criterion of the canonical form of a TBP-binding site [122]); K_{SLIDE} is the equilibrium constant of the first step of the TBP sliding along DNA; we estimated its value empirically as

$$-\ln(K_{SLIDE}) = MEAN_{15bp}\ \{0.8[TA]_{3'HALF} - 3.4MGW_{CENTER} - 35.1\},$$

where $[TA]_{3'HALF}$ is the abundance of dinucleotide TA within the 3′ half of the sequence being analyzed; MGW_{CENTER} is the mean width of the minor groove of the B-form of the DNA helix [125]; 0.8, –3.4, and –35.1 are linear regression coefficients calculated by means of the standard statistical package STATISTICA (Statsoft™, Tulsa, USA) from our original experimental data [129].

In Eq. **(1)**, K_{BEND} is the equilibrium constant at the third step of DNA helix bending; we estimated its value empirically as

$$-\ln(K_{BEND}) = MEAN_{TATA\text{-}box}\ \{0.9[TA, AA, TG, AG]_{FLANK} + 2.5[TA, TC, TG\ ]_{CENTER} + 14.4\},$$

where 0.9, 2.5, and 14.4 are linear regression coefficients calculated in STATISTICA from our original experimental data [130]; $MEAN_{TATA\text{-}box}$ is the mean value for both DNA strands of the TBP-binding site at the position of the maximal score value of Bucher's position-weight matrix [122].

According to all the 78 possible nucleotide substitutions, $s_{i+j}\rightarrow\xi$, at each j-th position ($-13 \le j \le 12$; 3×26) within the 26-bp DNA window centered by i-th position of the promoter DNA being analyzed, we estimated heuristically the standard deviation of the $-\ln[K_D]$ estimates (Eq. 1), namely:

$$\delta=[(\Sigma_{1\le i\le 26}\Sigma_{\xi\in\{a,c,g,t\}}[\ln(K_D(\{s_{i-13}\ldots s_{i+j-1}\xi s_{i+j+1}\ldots s_{i+12}\})/K_D(\{s_{i-13}\ldots s_{i+j-1}s_{i+j}s_{i+j+1}\ldots s_{i+12}\})^2])/78]^{1/2}. \qquad \textbf{(2)}$$

This equation **(2)** estimates the resistance to the majority of SNPs in the case of a biologically essential complex of TBP with the TBP-binding site of a promoter.

Applying the results of Eqs. **(1–2)** to the promoter DNA sequences of two minor and ancestral alleles of a given gene, $(-\ln(K_D^{(min)}) \pm \delta_{(min)})$ and $(-\ln(K_D^{(hg19)}) \pm \delta_{(hg19)})$, we calculated Fisher's Z-score as

$$Z=abs[\ln(K_D^{(min)}/K_D^{(hg19)})]/[\delta^2_{(min)}+\delta^2_{(hg19)}]^{1/2}.$$

The standard statistical package R [138] transformed this Fisher's Z-score value (as input data) into the p value (as output data), i.e., the probability rate of acceptance of the hypothesis "H_0: $-\ln(K_D^{(min)}) \neq -\ln(K_D^{(hg19)})$" (where $\alpha = 1 - p$ is the statistical significance level). At this statistical-significance level, we finally made a decision:

IF {$-\ln(K_D^{(min)})$ is statistically significantly greater than $-\ln(K_D^{(hg19)})$},

THEN {**DECISION** is "there is an excess of the minor allele of a given gene versus the ancestral allele"};

ELSE [**IF** {$-\ln(K_D^{(min)})$ is statistically significantly less than $-\ln(K_D^{(hg19)})$},

THEN {**DECISION** is "there is a deficiency of the minor allele of this gene versus the ancestral allele"},]

OTHERWISE {**DECISION** is "alteration of the expression of this gene is insignificant"}.
